# Neuregulin-1 Regulates Cell Adhesion via an ErbB2/Phosphoinositide-3 Kinase/Akt-Dependent Pathway: Potential Implications for Schizophrenia and Cancer

**DOI:** 10.1371/journal.pone.0001369

**Published:** 2007-12-26

**Authors:** Christopher G. Kanakry, Zhen Li, Yoko Nakai, Yoshitatsu Sei, Daniel R. Weinberger

**Affiliations:** 1 Genes, Cognition and Psychosis Program, Intramural Research Program, National Institute of Mental Health, National Institutes of Health (NIH), Bethesda, Maryland, United States of America; 2 Howard Hughes Medical Institute (HHMI)–National Institutes of Health (NIH) Research Scholars Program, Chevy Chase, Maryland, United States of America; University of Massachusetts Medical School, United States of America

## Abstract

**Background:**

Neuregulin-1 (NRG1) is a putative schizophrenia susceptibility gene involved extensively in central nervous system development as well as cancer invasion and metastasis. Using a B lymphoblast cell model, we previously demonstrated impairment in NRG1α-mediated migration in cells derived from patients with schizophrenia as well as effects of risk alleles in NRG1 and catechol-O-methyltransferase (COMT), a second gene implicated both in schizophrenia susceptibility and in cancer.

**Methodology/Principal Findings:**

Here, we examine cell adhesion, an essential component process of cell motility, using an integrin-mediated cell adhesion assay based on an interaction between ICAM-1 and the CD11a/CD18 integrin heterodimer expressed on lymphoblasts. In our assay, NRG1α induces lymphoblasts to assume varying levels of adhesion characterized by time-dependent fluctuations in the firmness of attachment. The maximum range of variation in adhesion over sixty minutes correlates strongly with NRG1α-induced migration (r^2^ = 0.61). NRG1α-induced adhesion variation is blocked by erbB2, PI3K, and Akt inhibitors, but not by PLC, ROCK, MLCK, or MEK inhibitors, implicating the erbB2/PI3K/Akt1 signaling pathway in NRG1-stimulated, integrin-mediated cell adhesion. In cell lines from 20 patients with schizophrenia and 20 normal controls, cells from patients show a significant deficiency in the range of NRG1α-induced adhesion (p = 0.0002). In contrast, the response of patient-derived cells to phorbol myristate acetate is unimpaired. The COMT Val108/158Met genotype demonstrates a strong trend towards predicting the range of the NRG1α-induced adhesion response with risk homozygotes having decreased variation in cell adhesion even in normal subjects (p = 0.063).

**Conclusion/Significance:**

Our findings suggest that a mechanism of the NRG1 genetic association with schizophrenia may involve the molecular biology of cell adhesion.

## Introduction

The predominant current view of the pathogenesis of schizophrenia posits that it is a neurodevelopmental disorder related to genetic factors that impact on brain development and plasticity.[1] Among the steadily increasing number of putative schizophrenia susceptibility genes is Neuregulin-1 (NRG1), which was first found to be associated with increased risk for schizophrenia in an Icelandic population[2] and subsequently confirmed in several studies of other populations.[3] NRG1 exists in multiple isoforms, all of which contain an epidermal growth factor (EGF)-like domain that can signal through erbB2, erbB3, or erbB4 receptors.[4] Most NRG1 isoforms exist as transmembrane proteins that are believed to be proteolytically cleaved, allowing the extracellular, EGF-like domain to bind to erbB receptors.[5] NRG1 is involved in many key neurodevelopmental functions, including neuronal migration, synaptic modulation, and oligodendrocyte development. [4], [6] Since these functions and others regulated by NRG1 are at least theoretically related to schizophrenia's putative neurodevelopmental origin, NRG1 is a biologically plausible schizophrenia susceptibility gene. Nevertheless, the specific biologic mechanism of its genetic association with schizophrenia and its role in disease pathogenesis are unknown.

The use of B lymphoblasts as a cell model to study basic molecular mechanisms and genetic associations of a number of human diseases, including neuropsychiatric disorders, is becoming increasingly widespread.[7], [8], [9] The application of this model to explore cell migration seems particularly appropriate given that neuronal and immune cells share many common molecular and cellular features important for cell motility.[10], [11], [12] Thus, molecular characterization and genetic associations of cell motility in cells of hematopoietic origin may provide insight into neuronal cellular processes. In recent work using a B lymphoblast cell model, we showed that B lymphoblasts express erbB2 and erbB3 receptors, and that NRG1α signals through these receptors via the PI3K/Akt and PLC pathways in order to promote chemotactic migration.[7] We also found that genetic variation both in NRG1 and COMT previously associated with schizophrenia predicted the migratory response of these cells to NRG1, suggesting a potential cellular mechanism for the genetic association to the disorder. In this study, we examine cell adhesion, a fundamental component process involved in cell motility. To do so, we have developed an integrin-mediated cell adhesion assay in 96-well plates based on an interaction between recombinant human ICAM-1 and its CD11a/CD18 integrin heterodimer receptor expressed on lymphoblasts.

Integrins are a class of cell surface receptors that play important roles in a vast array of cellular and physiological processes, ranging from embryonic development to immunity to tumor metastasis.[13], [14] Integrins form heterodimeric, transmembrane receptors that consist of an α subunit non-covalently associated with a β subunit. Integrins mediate signaling bidirectionally, helping to integrate intracellular processes with the extracellular environment. Attachment of integrins to extracellular matrix proteins or counterreceptors present on other cells activates so-called “outside-in” signaling that promotes a variety of cellular processes, including the regulation of cell survival, proliferation, and differentiation.[15] Meanwhile, chemokine and other chemical stimulation of cells can modulate the adhesiveness of integrins, promoting so-called “inside-out” signaling.[16], [17], [18], [19]

Integrins also have been implicated as playing key functions in mediating neuronal migration[20] and synapse formation and maintenance. They play critical roles in a host of neurodevelopmental processes, including neuronal precursor cell adhesion, chain migration, differentiation, and proliferation;[21], [22] regulation of neural-radial glial interactions and promotion of proper cortical laminar organization through interactions with Reelin;[23] correct migration of optic tectal neuroblasts into specific laminae;[24] proper neural crest development;[25] neural growth cone filopodial modulation;[26] oligodendrocyte survival;[27] and direction of neuromuscular synaptogenesis and migration of Schwann cells to the periphery.[28] Among their other roles in synapse biology are promotion of synaptic maturation,[29] mediation of synaptic plasticity,[30], [31] and crucial involvement in LTP induction[32] and stabilization[33], [34] as well as spatial memory.[32]

Because NRG1 and integrins converge on various developmental processes linked to neuronal migration and to synapse biology and given our previous findings related to NRG1-mediated cell migration, we explored the effects of NRG1 activation of lymphoblasts on integrin-mediated cell adhesion. We report that NRG1α induces varying levels of cell adhesion characterized by time-dependent fluctuations in the firmness of attachment as measured with a novel adhesion assay. The maximum range of this variation in adhesion over time (hereafter abbreviated as MRVA) correlates highly with the effectiveness of NRG1α-induced cell migration, indicating that the MRVA measure has biological relevance, at least to these cells. NRG1α-induced MRVA is dependent on erbB2/PI3K/Akt signaling. Furthermore, the MRVA induced by NRG1α is significantly lower in B lymphoblasts derived from patients with schizophrenia compared with those derived from normal controls. Lastly, a single nucleotide polymorphism within COMT, a gene implicated in susceptibility both to schizophrenia and to metastatic cancer, demonstrates a strong trend towards predicting the MRVA of a given cell line, even in normal subjects. Overall, our studies show that NRG1 signaling activates adhesion machinery in a process that is impaired in cells derived from patients with schizophrenia. If these findings in immune cells are conserved in neuronal cell processes, they would argue that a mechanism of the NRG1 genetic association with schizophrenia involves the molecular biology of cell adhesion.

## Results

### Lymphoblasts express integrins homogeneously

Lymphocytes predominantly express the CD11a/CD18 integrin heterodimer (α_L_/β_2_, which is also known as leukocyte function-associated antigen-1 or LFA-1).[35] We used flow cytometry to assay baseline integrin expression and to compare expression between cell lines derived from patients with schizophrenia versus normal controls. All tested cell lines expressed CD11a (corrected median fluorescence, 14.018±0.968) and CD18 (14.336±1.019), except for one patient-derived cell line. This cell line was excluded from all further testing and statistical comparisons. There was no significant difference between cell lines derived from patients or controls in the expression of either CD11a (patients (n = 23), 13.479±1.364; controls (n = 35), 14.790±1.303; p = 0.5046) or CD18 (patients (n = 23), 14.215±1.633; controls (n = 35), 14.832±1.291; p = 0.7667). Correlation of CD11a with CD18 expression (linear regression (n = 58), r = 0.806; Fisher's r to z, p<0.0001) was consistent with the conclusion that CD11a and CD18 principally were forming heterodimers with each other. Thus, these data demonstrate that our lymphoblast cell lines express the CD11a/CD18 integrins and that differential baseline expression of these integrins is not a major factor in differences in migration[7] or adhesion (see below) of patient-derived lymphoblasts.

### NRG1α induces temporally varying cell adhesion

**Figure 1:**
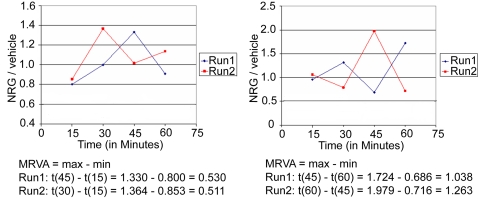
Neuregulin-1 induces temporally varying cell adhesion that appears characteristic of a particular cell line. In response to NRG1α stimulation over time, cells of a given cell line as a population fluctuate between states of relative detachment and states of relative attachment. In order to test the reliability of these patterns, ten cell lines on two separate days were tested using our adhesion assay. Two representative examples are shown. While the timing of peaks and troughs varied between runs, the maximum range of varying adhesion (MRVA) was remarkably consistent (p = 0.016). Calculation of the MRVA for each run of each cell line is shown.

Initial experiments with our adhesion assay suggested that adhesion had a time dependency. So, time course experiments were performed in which lymphoblasts were exposed to NRG1α or phorbol myristate acetate (PMA) in ICAM-coated plates for either 15, 30, 45, or 60 minutes, and then the plates were washed to remove cells that were not firmly adherent. PMA was used as a positive control as it promotes cell attachment by directly activating PKC. For each cell line, cells were subjected for the same time exposure to the ICAM-coated plates in the absence of NRG1α or PMA. Variation in the raw NRG1 attachment data (Figure S1) and in the NRG/vehicle ratio over time (Figure 1, Figure S1, Figure S2, and Figure S3) indicated that NRG1α-stimulated cells as a population were fluctuating between states of relatively firmer and weaker attachment compared with baseline control adhesion. In order to quantify the observed variation in cell adhesion, we defined the NRG1-induced maximum range of varying adhesion (MRVA) as the maximum NRG/vehicle ratio minus the minimum NRG/vehicle ratio of the four time points (Figure 1).

### The maximum range of varying adhesion (MRVA) appears characteristic of each cell line

To further address the reliability of these patterns, we performed repeated adhesion assays on ten cell lines on two separate days. These ten lines were selected to provide a spectrum of MRVA values in both patient and control cell lines, including high, middle, and low levels of variation. Clear patterns of varying adhesion over time were seen on both days, and though the actual pattern varied between repeat runs in terms of the specific times that defined the range of adhesion, the range of adhesion responses observed (i.e. the MRVA measure) was highly reproducible (Spearman rank correlation (n = 10), rho = 0.806, p = 0.016) (Figure 1).

### The MRVA of NRG1α-induced cell adhesion predicts NRG1α-induced migration

**Figure 2:**
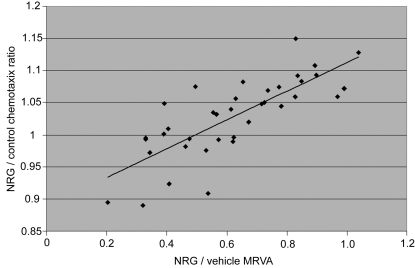
The maximum range of NRG1α-induced varying adhesion predicts NRG1α-induced chemotactic migration. Adhesion data were compared with available data gathered one year previously of NRG1α-induced migration of those same cell lines.

The MRVA correlated positively and highly significantly with the degree of chemotactic migration in response to NRG1α (linear regression (n = 37), r^2^ = 0.6065; Fisher's r to z, p<0.0001) (Figure 2), using migration data we have previously reported in the same cell lines.[7] (Three cell lines that were used for the adhesion studies lacked migration data and so were not included in this comparison.) These data suggest that the MRVA measure has broader biological significance, at least to these cells. Although correlation does not indicate causation, a varying adhesive state is a biologically plausible and necessary mechanism for cell migration.

### Defining the outcome measures

Given the reliability of the MRVA within a cell line and its apparent biological significance, the MRVA was established at this point as the primary dependent measurement for all future adhesion experiments. In addition, several other dependent measures were defined and used for studying the effects of signaling pathway inhibitors. Two other range measures were defined as follows: 1) ICAM-only MRVA, which is calculated from the control cells alone (maximum minus minimum of the four time points) and represents the effects of endogenous signaling and/or the presence of ICAM itself in modulating varying adhesion over time, and 2) PMA/vehicle MRVA, which is calculated analogously to NRG/vehicle MRVA and represents the variation in cell adhesion over time in response to PMA stimulation. A measure of net baseline attachment also was defined as follows: mean ICAM effect, which is the average of the four ICAM fluorescence values and represents the overall “stickiness” or relative adherence state of the control cells at baseline. In situations where an inhibitor exerted effects on both baseline and chemical-induced adhesion, the mean NRG and mean PMA fluorescence values were analyzed to distinguish effects on the numerator versus the denominator of the mean NRG/vehicle or mean PMA/vehicle ratios.

### NRG1α mediates its effects on cell adhesion through erbB2 signaling

**Figure 3:**
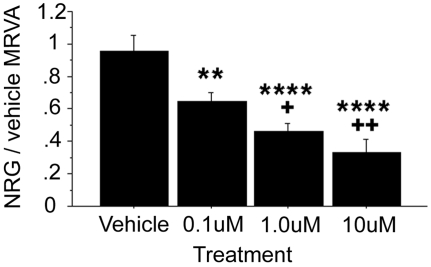
The selective erbB2 antagonist AG825 dampens the NRG1α-induced MRVA. Administration of AG825 in DMSO vehicle (n = 5) caused a robust, dose-dependent decrease in the MRVA induced by NRG1α. Compared with the vehicle control, ** represents p<0.01 and **** represents p<0.0001. Compared with the low dose, + represents p<0.05 and +++ represents p<0.001.

We previously have demonstrated that NRG1α-mediated migration of lymphoblasts was dependent on erbB2 signaling; thus, we tested whether erbB2 stimulation was involved in the mechanism of NRG1α-induced cell adhesion by treating cells with the selective erbB2 inhibitor AG825. AG825 exhibited a significant dose-dependent effect on dampening the MRVA of NRG1α-induced adhesion (ANOVA, F(3,4) = 23.358, p<0.0001) (Figures 3).

### PI3K signaling is essential for mediating NRG1α-induced varying cell adhesion

**Figure 4 pone-0001369-g004:**
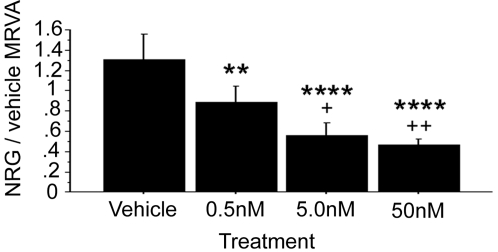
The phosphoinositide-3 kinase (PI3K) inhibitor wortmannin is essential for variation in NRG1α-induced cell adhesion. Wortmannin in DMSO vehicle (n = 5) exhibited a strong, dose-dependent, dampening effect on the NRG/vehicle MRVA measure. Compared with the vehicle control, * represents p<0.05, ** represents p<0.01, and **** represents p<0.0001. Compared with the low dose, + represents p<0.05 and ++ represents p<0.01.

To address the role of PI3K signaling in the NRG1α-induced adhesion effect, we studied the irreversible PI3K inhibitor wortmannin. The doses used were all well below concentrations in which wortmannin cross-reacts with MAPK, myosin light chain kinase (MLCK), or phosphoinositide-4 kinase signaling in an effort to specifically target PI3K signaling. Wortmannin exhibited a significant, dose-dependent, dampening effect on the NRG/vehicle MRVA (ANOVA, F(3,4) = 17.208, p<0.0001) (Figure 4) with no effects on baseline or PMA-induced adhesion.

### Akt inhibition mimics PI3K inhibition and dampens the NRG1α-induced adhesion effect

**Figure 5 pone-0001369-g005:**
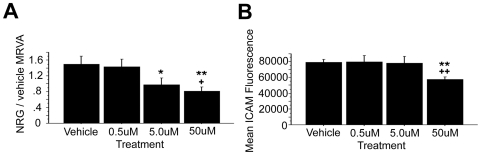
Akt inhibition causes a dampening of the NRG/vehicle MRVA measure. Administration of the Akt Inhibitor X in water vehicle (n = 5) caused a dose-dependent decline in the NRG/vehicle MRVA (A). Unlike PI3K inhibition, Akt inhibition was associated with a statistically significant reduction in mean ICAM (B) and mean NRG (not shown) raw fluorescence values at the high dose only with no trend at lower doses. Compared with the vehicle control, * represents p<0.05 and ** represents p<0.01. Compared with the low dose, + represents p<0.05 and ++ represents p<0.01.

The Akt Inhibitor X selectively inhibits the phosphorylation of Akt with minimal effects on PI3K, PDK1, or SGK1 signaling. The effects of this drug mirrored the results of PI3K inhibition with a dose-dependent, significant reduction in NRG/vehicle MRVA (ANOVA, F(3,4) = 4.709, p = 0.0214) (Figure 5a) and no effects on ICAM-alone or PMA/vehicle MRVA. Unlike with PI3K inhibition, Akt inhibition was associated with a significant reduction in mean ICAM-alone attachment (ANOVA, F(3,4) = 6.711, p = 0.0086) (Figure 5b). However, this reduction was only seen at the highest dose (p = 0.0033). This same effect also was seen in significantly reducing the raw NRG fluorescence values at the high dose only (ANOVA, F(3,4) = 11.639, p = 0.0007) with no obvious trend at other doses. Given this common effect on mean ICAM-alone and mean NRG attachment exclusively at the high dose, it may represent a cross-reaction of the inhibitor at high concentrations with another signaling pathway important for maintaining an adhesive state.

### Rho kinase is not essential for the NRG1- or PMA-induced adhesion effects

Since Rho also is downstream of PI3K and has been implicated in cell adhesion mechanisms,[36], [37] it was important to investigate whether Rho plays a role in NRG1-induced cell adhesion. However, there are no cell-permeable, specific Rho inhibitors available, so the Rho-associated protein kinase (ROCK) inhibitor Rho Kinase Inhibitor IV was used. This ROCK inhibitor had no significant effects on any of adhesion parameters examined, and so ROCK signaling does not appear to play any critical role in modulating baseline, NRG1-induced, or PMA-induced varying adhesion.

### MEK signaling is not essential for the NRG1-induced cell adhesion effect

The selective MEK inhibitor PD98059 had no effect on any of the adhesion parameters at any dose (not shown). This suggests that even were MEK signaling involved in these processes, it is not essential for baseline adhesion or NRG1- or PMA-mediated adhesion.

### Phospholipase C signaling is necessary for maintaining an adhesive state in general

**Figure 6 pone-0001369-g006:**
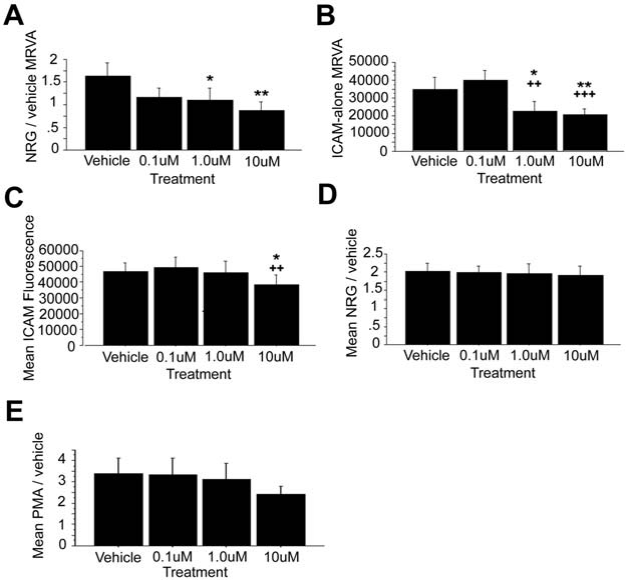
Phospholipase C (PLC) is essential for maintaining an adhesive state in general. While the PLC inhibitor U73122 in ethanol vehicle (n = 5) did cause a significant reduction in NRG/vehicle MRVA (A) and a trend towards reduction in PMA/vehicle MRVA (not shown), it exerted a much stronger effect on dampening baseline MRVA due to the presence of ICAM and endogeneous signaling alone (B). Furthermore, at high dose, it caused a significant reduction in baseline ICAM attachment (C) and in mean NRG and PMA attachment (raw fluorescence values) (not shown). However, mean NRG/vehicle (D) and mean PMA/vehicle (E) were not significantly changed. Compared with the vehicle control, * represents p<0.05 and ** represents p<0.01. Compared with the low dose, ++ represents p<0.01 and +++ represents p<0.001.

Like PI3K, the PLC inhibitor U73122 also caused a significant reduction in the NRG1α-induced MRVA (ANOVA, F(3,4) = 4.186, p = 0.0304) (Figure 6a), but the effect of this inhibitor seemed to be an overall reduction in cell attachment in general, and not an NRG1/erbB2-dependent effect. Both baseline variability (ICAM-alone MRVA) (ANOVA, F(3,4) = 8.883, p = 0.0023) and baseline mean attachment (mean ICAM-alone) (ANOVA, F(3,4) = 4.915, p = 0.0186) decreased after exposure to U73122 (Figures 6b and c, respectively). However, the change in mean ICAM-alone was only significant at the highest dose (p = 0.0155) (Figure 6c). Likewise, the raw mean NRG and PMA fluorescence values had significant decreases only at the highest dose (NRG: ANOVA, F(3,4) = 8.672, p = 0.0025; PMA: ANOVA, F(3,4) = 4.736, p = 0.0210). It is important to note, however, that neither the mean NRG/vehicle (Figure 6d) nor the mean PMA/vehicle (Figure 6e) ratios were significantly different across doses, indicating that PLC inhibition is affecting common cellular signaling across these pathways to a similar degree independent of the stimulatory agent. Therefore, although the PLC inhibitor did exert a significant effect on reducing the NRG/vehicle MRVA, this likely was due to reducing overall adhesion and constricting the range of the potential response, rather than to specific inhibition of an NRG1/erbB2-dependent pathway.

### Myosin light chain kinase (MLCK) signaling is important for baseline adhesion

**Figure 7:**
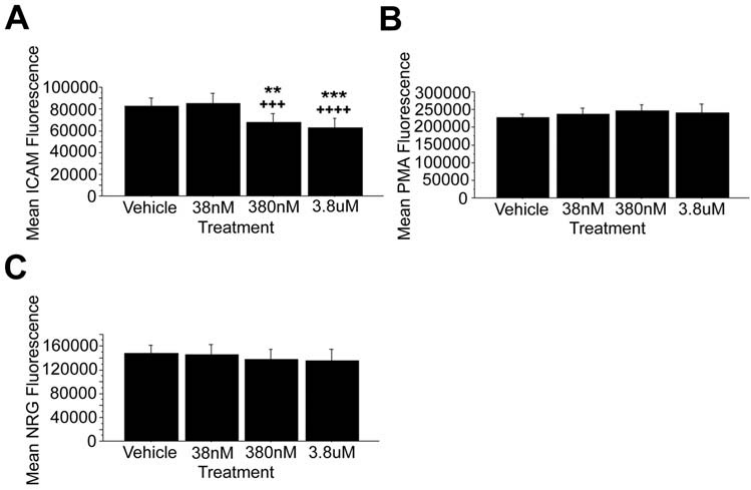
Myosin light chain kinase (MLCK) is important for baseline attachment. Administration of the MLCK inhibitor ML-7 in DMSO vehicle (n = 5) caused a significant reduction in baseline attachment at the middle and high doses (A). Although this was associated with increases in mean NRG/vehicle and mean PMA/vehicle (not shown), these effects were merely a consequence of reductions in the denominator as both the raw PMA (B) and NRG (C) fluorescence values were unchanged. Compared with the vehicle control, ** represents p<0.01 and *** represents p<0.001. Compared with the low dose, +++ represents p<0.001 and ++++ represents p<0.0001.

Given the known importance of MLCK in promoting attachment, the MLCK inhibitor ML-7 was used to examine its effects on our various adhesion parameters. As expected, the main effect of MLCK inhibition was a dose-dependent decrease in mean ICAM-alone attachment (ANOVA, F(3,4) = 17.601, p<0.0001) (Figure 7a). However, there were no changes in the raw fluorescence values of PMA (ANOVA, F(3,4) = 0.381, p = 0.7686) (Figure 7b) or NRG (ANOVA, F(3,4) = 0.322, p = 0.8095) (Figure 7c). No significant effects on any of the three MRVA parameters were seen. It is also important to highlight that the effects observed by PLC versus MLCK inhibition were not identical. PLC inhibition caused decreases in the raw NRG and PMA fluorescence values, suggesting its broad importance to multiple common pathways of adhesion. In contrast, MLCK inhibition seemed to exert an effect restricted to baseline ICAM adhesion with no effects on the raw mean NRG or PMA fluorescence values.

### NRG1α-induced varying adhesion is dampened in lymphoblasts derived from patients with schizophrenia

**Figure 8:**
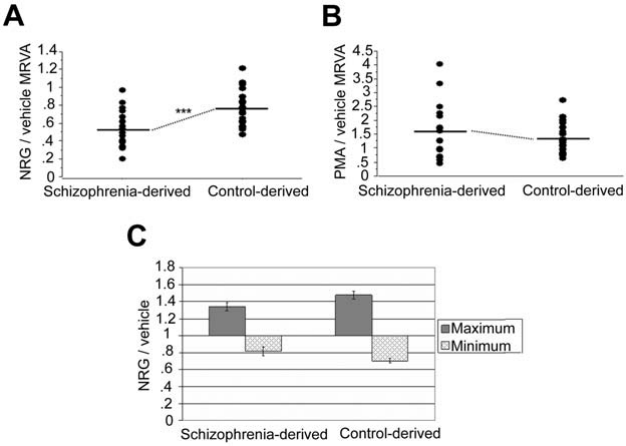
Neuregulin-1-induced, integrin-mediated varying cell adhesion is impaired in cell lines derived from patients with schizophrenia. The NRG/vehicle MRVA was significantly lower in lymphoblasts derived from patients compared with those derived from normal controls (A). PMA/vehicle MRVA was similar between patient-derived and control-derived cell lines (B). The relative impairment in patient-derived cells did not seem to be isolated to attachment or detachment, but was indicative of an overall more constricted response with less variation from baseline adhesion in response to NRG1α (C). *** represents p<0.001.

Given our prior observation that patient-derived lymphoblasts showed a less robust migratory response to NRG1α and the current correlation between our migration and adhesion measures, we predicted that there would also be a MRVA difference between patients and controls. Indeed, the NRG/vehicle MRVA was significantly lower in lymphoblasts derived from patients compared with those derived from normal controls (patients (n = 20), 0.520±0.045; controls (n = 20), 0.780±0.044; p = 0.0002) (Figure 8a and c). In contrast, the PMA response was similar between groups, indicating that the patient-derived lymphoblasts still retained the ability to mediate robust cell adhesion, but were unable to do so appropriately in response to NRG1α. A series of PMA dose-response experiments were conducted to rule out the possibility that the lack of differences between groups was a dose-sensitive phenomenon; these tests showed nearly identical wave forms in all subjects at all assayed concentrations (Figure S2), further suggesting that the PMA response was normal in the patient-derived cell lines. It is important to note that PMA also induced varying levels of adhesion in our assay, similar to but more extreme than that of NRG1α. Both the MRVA measure of PMA-induced adhesion (patients (n = 20), 1.561±0.217; controls (n = 20), 1.356±0.121; p = 0.4124) (Figure 8b) and the mean PMA/vehicle (patients (n = 20), 2.083±0.120; controls (n = 20), 1.980±0.085; p = 0.4840) did not differ between patient-derived and control-derived cells. There was no difference in mean ICAM attachment between patient-derived and control-derived cells in the unstimulated ICAM-alone condition.

### COMT genotype predicts the range of variation in NRG1α-induced adhesion

**Figure 9:**
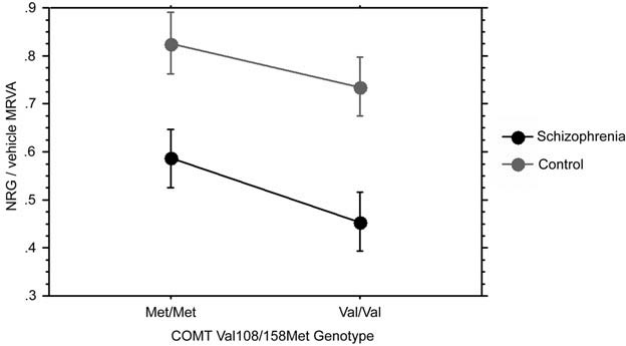
COMT Val108/158Met predicts variation in cell adhesion. COMT, a putative susceptibility gene for both schizophrenia and metastatic cancer, previously was shown to have genotype effects on cell migration with the risk genotype rs4680 V/V showing the most impaired migration. In our adhesion data (n = 40), there was a strong trend (p = 0.0632) towards decreased NRG1α-induced MRVA of risk homozygotes compared with non-risk homozygotes (no heterozygote cell lines are maintained).

Given the relationship of single nucleotide polymorphisms in COMT (val108/158met, rs4680) and in NRG1 (rs6994992) to NRG1α-induced cell migration established previously,[7] we sought to determine if NRG1α-induced cell adhesion also showed such associations. The presence of the schizophrenia risk-associated COMT val allele showed a strong trend towards predicting smaller NRG1α-induced MRVA in the entire sample with similar effects in both groups (unpaired t-test, p = 0.0632) (Figure 9a). ANOVA performed with diagnostic group and genotype as independent factors revealed no genotype by diagnosis interactions for either gene. Additionally, there was no effect of COMT genotype on PMA-induced MRVA (unpaired t-test, p = 0.8727). Because of the low minor allele frequency at NRG1 rs6994992, our study was underpowered to test for an association with this SNP.

## Discussion

We have presented a series of studies that provide evidence that NRG1α activation of B lymphoblasts induces a temporally varying cell adhesion response which is mediated by the erbB2/PI3K/Akt1 pathway and which is abnormal in cells derived from patients with schizophrenia and affected by COMT val/met genotype. To our knowledge, these are the first data showing that NRG1α induces attachment in B lymphoblasts in an assay of cell adhesion, revealing time-dependent fluctuation in net adhesion. In our studies, the MRVA of this NRG1α-induced adhesion response has apparent biological relevance in that it predicts both the degree of the cell migration response to NRG1α as well as differences between schizophrenia cases and normal control subjects. In turn, the MRVA itself may be predicted by a single nucleotide polymorphism within COMT, which is also implicated in metastatic cancer. While exogenous NRG1α increases the cell surface expression of the CD18 integrin (Figure S4), the stable nature of this effect over time suggests that this increased surface expression is not directly responsible for mediating the temporally varying adhesion response. Rather, this NRG1α-induced adhesion response is dependent on signaling through erbB2, PI3K, and Akt pathways. PLC and MLCK signaling also play roles in adhesion, but do not exert NRG1α-specific effects.

### Methodological considerations

As the time-dependent variation in cell adhesion in response to NRG1 is the critical phenomenon upon which the work reported herein is based, it is important to validate that this variation represents a real biological effect and is not merely “noise.” Indeed, it could be argued that since our measure is based on a population of cells that likely is not homogeneous with respect to attachment states, cell cycle phases, and other biological phenomena, the MRVA is more likely random than not. We have performed a series of detailed experiments showing convincingly and consistently that this varying adhesion is not random: specifically, 1) particular temporal patterns of attachment and detachment, while not intrinsic to the unstimulated cell itself, seem to be relatively conserved when cells from the same cell line under the same conditions at the same time are subjected to specific stimuli which change their attachment state (Figure S2); 2) the MRVA is remarkably reliable within a cell line studied on repeated occasions on different days (Figure 1); 3) the MRVA is affected in a dose-dependent manner by various relevant signaling pathway inhibitors (Figures 3–7); 4) the MRVA induced by NRG1 predicts other NRG1-related phenomena in these cells, e.g. NRG1-induced migration (Figure 2), in contrast to the PMA-induced MRVA measure, which predicts none of these effects; and 5) the NRG1-induced MRVA is predicted by variation in COMT, and the pattern of this relationship is similar in patients with schizophrenia and in normal subjects (Figure 9). We would not expect to find such systematic properties were the effects simply noise. On the contrary, we suspect that this varying level of net adhesion represents, at the individual cell level, an oscillatory phenomenon related to the cell biology of motility, though we are unable to confirm mathematically the oscillatory nature of this phenomenon at this point.

A second potential concern involves the constraints of the time course experiment itself. In the literature, investigators studying cell adhesion generally look at one specific time point (often 1 hour) as the outcome measure. As our initial studies indicated that NRG1-induced adhesion actually had a time dependency, we chose four evenly spaced time points over that hour to better characterize the varying nature of this adhesion. More time points might be preferable in order to better assess the precise variations that are occurring. Unfortunately, technical and other considerations make assaying at more frequent time points impractical. Theoretically, cell adhesion could be fluctuating with a period on the order of seconds or even more quickly. It is unclear whether such effects would be observable in a population of cells even with continuous sampling, which is not possible with our assay. These considerations notwithstanding, the fact that there are observable mass effects suggests that the cells have a certain degree of synchronization in their modulation of adhesion or else no net effect over time would be expected. This synchronization is not entirely surprising given that all cells likely are starting at roughly the same basal adhesive state, due to similar cell and culture conditions, and then are being exposed to the same exogenous dose of NRG1α. Cell to cell interactions also likely play a role in mediating this mass effect. Nevertheless, the MRVA as described does serve to quantify the range of the adhesion over time in a cell line and is closely related to NRG1α-induced cell motility. The fact that our MRVA measure and our measure of NRG1-induced motility were highly correlated even though the experiments were performed on average 13 months apart, suggests that these are biologic characteristics of a cell line and not artifacts of cell preparation or culture conditions.

Another potential confounder for which it is difficult to control is the possible effect of endogenous NRG1. It has recently been shown that B lymphoblasts do not express NRG1,[38] though the serum in the culture medium likely does contain NRG1. So, even the cells in the control wells are likely being exposed to low levels of NRG1, although the relative levels of stimulation are different from exposure to exogenous NRG1. Pre-exposure to low levels of serum-derived NRG1 may contribute to baseline ICAM-alone MRVA. However, assuming that this serum-derived NRG1 effect is stable over time and independent of exogenous NRG1 stimulation, the fact that we are always comparing NRG1-stimulated cells to control cells controls for any net effects on adhesion that serum-derived NRG1 exerts.

The last methodological consideration relates to the form of NRG1 employed in these studies. While the β form is more potent and the more widely available form in the central nervous system,[4] the α form is the type more commonly found in peripheral tissue. We elected to use the α form based on our peripherally derived cell type. Studies comparing the relative effects of the β versus the α form would be of interest.

### Integrin-mediated adhesion and cell signaling

The intracellular signaling pathways mediating integrin inside-out signaling are complex and yet to be fully clarified. Though several pathways have been implicated in this inside-out signaling, all seem to operate by one or both of two mechanisms: 1) changing integrin affinity, at least in part, through phosphorylation of integrin cytoplasmic domains, which can mediate conformational changes itself or which may lead to recruitment of other molecules that help mediate these processes, and 2) altering integrin clustering,[39] which affects integrin avidity and is dependent on calcium signaling[40] and the presence of lipid rafts.[41]

Phorbol esters, such as PMA, can promote integrin attachment by directly activating PKC independent of diacylglycerol.[42] They appear to do so, at least in part, through PKC phosphorylation of the β2 integrin and consequent recruitment of 14-3-3 protein to the activated integrin.[43] However, the importance of the PKC pathway for integrin activation, especially in response to chemokine signaling, is unclear since inhibition of the PKC pathway does not abolish chemokine-induced integrin activation.[42], [44], [45] On the other hand, the PI3K pathway appears to play a fundamental role in modulating cell adhesiveness through chemokine-induced inside-out signaling pathways.[46], [47], [48], [49] The PI3K pathway seems to mediate this signaling via regulation at the integrin cytoplasmic domains.[50] Rho, which is downstream of PI3K, inhibits clustering of LFA-1 molecules,[37] and the leukocyte-specific RhoH has been implicated as important in maintaining a nonadhesive state.[36] However, this picture is complicated by the fact that ROCK promotes myosin light chain phosphorylation,[51] which leads to increased attachment. In fact, T lymphocyte migration involves close regulation of MLCK-mediated attachment and ROCK-dependent detachment.[52] Additionally, intracellular calcium fluxes play a role in modulating cell adhesion through a variety of molecules, including PKC, MLCK, calmodulin,[53] calpain,[40] calreticulin,[54] and focal adhesion kinase.[55]

Consistent with both the literature[56] and our previous migration studies, we found that PI3K but not MEK is critical for mediating NRG1-induced adhesion. Our demonstration of strong non-specific effects of PLC in maintaining an adhesive state is consistent with the literature, especially given the known importance of both PKC and calcium signaling in promoting cell adhesion. Additionally, the role of MLCK in maintaining an adhesive state was confirmed by our studies. The lack of effect of ROCK inhibition on our adhesion studies was unexpected. However, given ROCK's particular roles in promoting both pro-adhesive and anti-adhesive pathways through effects on MLCK and Rho, respectively, the lack of a robust directional effect on cell adhesion may be due to complex compensatory mechanisms in these pathways. Finally, it recently has been demonstrated that Akt1 plays an important role in integrin activation through an inside-out pathway, emphasizing a regulatory role for Akt1 in cell adhesion and migration.[57] Our data not only help support these findings but may provide a potential mechanism towards understanding these regulatory effects.

While we demonstrated differences in our adhesion assay between our patient-derived and control-derived cells, the molecular basis of this difference is not yet identified. Our results could be specific to NRG1-mediated adhesion; they could reflect diverse sites of molecular pathology in NRG1 signaling related to the erbB/PI3K/Akt pathway; or the data could be related to more general phenomena that converge on cell adhesion. At present, the weight of the evidence suggests that patient-derived lymphoblasts do not have an intrinsic impairment of either cell attachment or detachment, but rather seem unable to modulate their adhesive states from their baseline levels in response to NRG1α.

### Potential implications for clinical disease

The strong relationship between the NRG1-induced adhesion response and the ability of the cells to migrate has potential implications for several clinical disease entities, most notably cancer and schizophrenia. Our data may have relevance for cancer biology, especially for B cell leukemia/lymphoma, multiple myleloma, and breast and other ErbB2-related cancers. NRG1 also been shown to be sufficient in promoting invasion[58] and metastasis[59], [60] of cancer cells, processes which are dependent on at least some of the same adhesion mechanisms. Furthermore, Akt activation has been found to be associated with NRG1-producing stromal cells and was a negative prognostic marker that was associated with increased risk of distant metastasis.[61]

While our studies do not show that the NRG1-induced adhesion response directly relates to these clinical phenotypes, they provide a consistent and biologically plausible mechanism that might help explain them and might be of value in studying them. If NRG1/erbB signaling in the B lymphoblast proves to be a good model for understanding aspects of NRG1-mediated neuronal adhesion and migration, then our findings may also provide insight into better understanding molecular and genetic mechanisms of schizophrenia, in which abnormalities of neuronal migration and synaptic connectivity have been implicated.[1]

Though we currently have no solid data to explain the mechanism of the association with COMT, we suggest that it reflects the impact of a by-product of this methyltransferase enzyme on cell motility. It has recently been found that s-adenosylhomocysteine hydrolase (SAHH) is localized at the front leading edge of migrating cells, suggesting that the endogenous methyltransferase inhibitor s-adenosylhomocysteine (SAH) needs to be actively removed at the leading edge[62]. This finding supports earlier observations that phospholipid methylation occurs at a rapid turnover rate during cell migration[63] and that migration is affected substantially by concentrations of s-adenosylmethionine (SAM) and SAH[64], [65]. COMT activity consumes SAM and generates SAH. COMT val alleles have about twice the enzyme activity of met alleles[66], predicting that they would generate relatively more SAH. Thus, it is possible that the COMT val/met polymorphism affects phospholipid methylation by modifying the SAM/SAH ratio, with val alleles thus having a lower ratio and poorer adhesion/migration.

It is also interesting to note that COMT is implicated in cancer biology and metastasis, and the allelic directionality is consistent with our results (i.e. here met alleles are risk-associated)[67]. Finally, our adhesion studies and findings have brought at least three promising schizophrenia susceptibility genes–NRG1, COMT, and Akt1–together in a coherent, biologically plausible framework. Not only does this underscore the polygenic complexity of cell adhesion and migration, but it provides a possible mechanism for the risk towards schizophrenia that these particular susceptibility genes confer.

## Materials and Methods

### Subjects

Blood collection and transformation of lymphocytes were approved by the NIMH institutional review board, and all donors gave written, informed consent. The B lymphoblast cell lines were gathered separately in two cohorts. The first cohort was derived from 15 normal controls (8 females, 7 males; age 34.9±2.4 years at the time of blood collection) and 13 individuals with schizophrenia (6 females, 7 males; age 36.3±3.6 years). A second cohort of B lymphoblasts was derived from 20 normal controls (10 females, 10 males; age 29.7±2.2 years) and 11 individuals with schizophrenia (5 females, 6 males; age 38.7±3.5 years). All subjects were drawn from individuals participating in the Clinical Brain Disorders Branch “Sibling Study” protocol, an ongoing investigation of neurobiological abnormalities related to genetic risk for schizophrenia. The details of subject recruitment and examination are described elsewhere.[68] Only Caucasian subjects of self-reported European ancestry were included to avoid genetic stratification and to reduce heterogeneity. Because of our prior interest in studying the effects of the COMT val108/158met polymorphism on various phenotypes, B lymphoblasts were selected only from val or met homozygous individuals for both case and control groups to increase statistical power for comparison. While every available cell line (n = 59) was assayed for baseline integrin expression, a total of 40 cell lines was tested using our adhesion assay to provide an equal number in the following categories: 1) schizophrenia, COMT val/val genotype; 2) schizophrenia, COMT met/met; 3) normal control, COMT val/val; 4) normal control, COMT met/met. These cell lines are derived from the same individuals described in our report on cell migration,[7] but represent cultures of transformed cells grown months apart from different frozen pellets of the original stock. For the cell lines used for these studies, patients and controls did not differ significantly in age (p = 0.5331 for total sample), gender, or race in either of the cohorts or in the total sample. For the pharmacological assays, each inhibitor was tested on five control cell lines, and the cell lines used for these assays varied based only on the availability of cells at the time of testing.

### B lymphoblast culture

Mononuclear cells were isolated by Ficoll-Hypaque density gradient centrifugation. B lymphocytes in the mononuclear cell preparation were transformed by infection with EBV as previously described.[69] The transformed B lymphoblasts were grown in RPMI-1640 medium (Gibco, Grand Island, NY) containing L-glutamine (2 mmol/L) (Gibco), 10% fetal bovine serum (FBS) (Cambrex, Walkersville, MD), 100 µg/mL streptomycin, and 100 units/mL penicillin (Gibco) in an incubator (95% air/5% CO2 at 37°C). The same lot of medium was used for all experiments. Cell lines periodically are recovered from the original stock every 3 months.

### Flow cytometric analyses of integrin expression

For the baseline integrin expression experiments, 0.5 million cells were stained with either FITC anti-human CD11a (BD Pharmingen, San Diego, CA) or FITC anti-human CD18 (BD Pharmingen). The same number of cells also was stained with FITC anti-mouse IgG_1_ (BD Pharmingen) for use as a negative control. For each integrin and cell line, the median fluorescence values were taken from gated regions of 10 000 live cells using a FACScan (Becton Dickinson, Franklin Lakes, NJ). A corrected median fluorescence was calculated by subtracting the negative control median fluorescence from the integrin median fluorescence. For the experiments looking at integrin expression following stimulation with recombinant human NRG1α (R&D Systems, Inc, Minneapolis, MN), 0.5 million cells were stained with either FITC anti-human CD11a or PE anti-human CD18 (BD Pharmingen). Unstimulated cells from the same cell lines were used as controls.

### Cell adhesion assay

Recombinant human ICAM-1 (ICAM) (R&D Systems) was adsorbed at 10 µg/ml (1 µg/well) to black, non-treated, 96-well plates (Nalge Nunc International, Rochester, NY) overnight at 4°C. Nonspecific adhesion was blocked with 3% bovine serum albumin (BSA) (Sigma-Aldrich, Allentown, PA) in phosphate buffered saline (PBS) for 2 hours at 4°C. Plates were washed 3 times each with PBS to remove nonadsorbed ICAM and BSA. 50 000 cells/well were added in triplicate to the plates, which were preloaded with control buffer (Hanks' Buffered Salt Solution (HBSS) with calcium and 10 mM HEPES), 50 ng/ml recombinant human NRG1α (R&D Systems) in control buffer, or 150 nM phorbol myristate acetate (PMA) (Sigma-Aldrich) in control buffer. The cells were allowed to incubate at 37°C for 15, 30, 45, or 60 minutes. At each of these time intervals, the plates were washed gently 4 times each with HBSS containing calcium using a 12-tip multi-pipettor. 150 µl PBS and 50 µl CyQuant GR dye/lysis buffer (Invitrogen, Carlsbad, CA), which fluorescently labels DNA, were added to each well. Plates were incubated in the dark on an orbital shaker at 100 rpm for 15 minutes and then read 5 times each using a Victor3 1420 Multilabel Counter fluorescence microplate reader (Perkin Elmer, Wellesley, MA). The read-out, therefore, was fluorescence-based quantification of the DNA of the cells that remained attached, and thus served as a proxy to quantify the number of adherent cells. We also performed a series of studies in two cell lines with five minute sampling rates (Figure S3). While this approach allowed a more complete characterization of the range of varying adhesion, it revealed no evidence of a regular pattern over this time period. Thus, we concluded that sampling noise would not be systematically affected by the 15 minute frequency, which was much less labor intensive and more practical for the full sample.

In a given experiment for the non-pharmacological inhibitor studies, four cell lines were assayed as a batch. A different plate was used for each time point. The placement of cell lines on each plate was varied such that cell lines derived from patients with schizophrenia versus normal controls were in different positions in different experiments, but in any given individual experiment, both patient-derived and control-derived cell lines were used. While the investigator was not blinded as to the plate arrangement, the washings were done with a 12-tip multi-pipettor such that wells containing different cell lines derived from individuals with and without the diagnosis of schizophrenia were washed at the same time and with the same technique. For the pharmacological assays, one cell line was assayed per experiment. For these studies, the plates were arranged such that the unstimulated wells from all treatment groups were washed at the same time and with the same technique as were the NRG1 wells and the PMA wells.

The effect of NRG1α stimulation was measured at each time point based on calculation of a ratio (NRG/vehicle) (Figure S1), which was determined by taking the mean of the triplicate NRG1α-stimulated fluorescence values and dividing by the mean of the triplicate of the control ICAM-alone fluorescence values. This normalization procedure was adopted because raw baseline adhesion states tended to show some drift over time, albeit much less than the pronounced variation in adhesion after NRG1 stimulation (Figure S1). Thus, the use of this ratio helps to control for other cellular and environmental processes that may impact adhesion. An analogous procedure was done to determine the effect of PMA stimulation, based on a PMA/vehicle ratio at each time point.

Dose response curves for NRG1α and PMA were performed across five NRG1α doses from 1–200 ng/ml and across five PMA doses from 1.5–300 nM (not shown). NRG1α had a slight peak in the MRVA around 50 ng/ml, so all cell lines were subjected to this concentration for all experiments. The PMA response was similar at commonly used dosages in the literature (100–300 nM) as well as at doses 100 times below those concentrations, so 150 nM was arbitrarily selected for the positive control as it was a modest dose relative to the literature.

For signaling pathway inhibitor pharmacological assays, cells were incubated with the particular inhibitor in the dark at room temperature for 40–60 minutes prior to plating. The inhibitors (AG825, wortmannin, Akt inhibitor X, Rho kinase inhibitor IV, PD98059, U73122, and ML-7) were obtained from Calbiochem, San Diego, CA. Unstimulated control cells for these experiments were incubated with equal volume of vehicle containing the particular inhibitor. Concentrations for inhibition were chosen by the middle concentration being approximately at the average IC_50_ reported in the literature (references per manufacturer's catalog). High and low doses were 10 times greater and 10 times less, respectively, than the middle dose.

### Statistical Analyses

Between groups comparisons were performed by unpaired t-tests or factorial ANOVA, where applicable. Effects of NRG1α on integrin expression and effects of signaling inhibitors both were analyzed by one-way repeated measure ANOVA to compare the dependent measures of the treated versus untreated cells. The effect of COMT genotype on the NRG/vehicle MRVA was assessed by one-tailed group comparison. All ANOVAs were followed by Fisher's post-hoc comparison matrices. Unless otherwise stated, data were expressed as mean +/− standard error of the mean (SEM).

## Supporting Information

Figure S1 Variation of attachment induced by NRG1 and PMA. The NRG/ICAM and PMA/ICAM values were designed to isolate the effects of NRG1α or PMA on the cell adhesion state and thereby control for the vast array of other cellular and environmental processes that can impact adhesion. These data illustrate that the use of such ratios is a justified transformation of the data and that the variable adhesion states are not an artifact simply of changing ICAM-alone baseline adhesion. Displayed are raw fluorescence values of ICAM-alone, NRG, and PMA data from three experiments on different days using different cell lines. (A, C, E). These data show that although baseline adhesion shows little variation over time, NRG and PMA adhesion do considerably. The NRG/vehicle and PMA/vehicle ratios for those same three experiments also are shown next to their respective raw data (B, D, F).
(0.85 MB TIF)

Figure S2 Varying adhesion patterns show individual consistency. Cells from three cell lines were subjected in the same experiment at the same time to three doses of PMA over two orders of magnitude (A-C). Wells from the three different cell lines were washed together at the same time and with the same technique using a 12-tip multi-pipettor. Within a cell line, the same general pattern of varying cell adhesion over time was conserved. However, the patterns of varying cell adhesion were quite different between cell lines with peaks and troughs occurring at different time points despite undergoing washings together at the exact same time with the multi-pipettor. This suggests that the response of cells from a particular cell line under the same conditions is relatively synchronized and that the varying adhesion observed is less likely to be simply noise.
(0.34 MB TIF)

Figure S3 The MRVA appears intrinsic to a particular cell line and is dampened upon inhibition of involved signaling pathways. A representative example of one control cell line assayed at 5 minute time points with and and without PI3K inhibition by wortmannin. While there is no predictable pattern, the vehicle cells continue to recapitulate the same approximate peak and trough multiple times over the time course. The MRVA for the wortmannin-treated cells is substantially lower than the MRVA for cells treated only with DMSO vehicle (wortmannin MRVA: 0.779, vehicle MRVA: 1.211). As the cells can fluctuate over a 5 minute period from states of relatively strong net attachment to states of relatively strong net detachment, there is no obvious advantage to using 5 minute time points instead of 15 minute time points in order to help characterize the range of variability. All wells for each time point were washed at the same time and with the same technique using a 12-tip multi-pipettor.
(0.33 MB TIF)

Figure S4 CD18, but not CD11a, integrin surface expression increases with NRG1α stimulation. Cells from 5 cell lines derived from patients with schizophrenia and 5 derived from normal controls were stimulated with NRG1α for 0, 15, 30, 45, or 60 minutes and then stained with anti-CD11a (αL) FITC-conjugated (A) or anti-CD18 (β2) PE-conjugated (B) antibodies and analyzed for expression by FACScan. The median fluorescence of 10 000 gated live cells was calculated for each cell line. Group data for both figures reflect the means of individual cell lines' median fluorescence values. *** represents p<0.001 and is the post-hoc value for every 2-way comparison with t = 0.
(0.10 MB TIF)
