# Supplementary figures and images for: Neuregulin-1 Regulates Cell Adhesion via an ErbB2/Phosphoinositide-3 Kinase/Akt-Dependent Pathway: Potential Implications for Schizophrenia and Cancer
Source: PLoS One. 2007 Dec 26;2(12):e1369. doi: 10.1371/journal.pone.0001369 (PMC2147048; doi:10.1371/journal.pone.0001369)

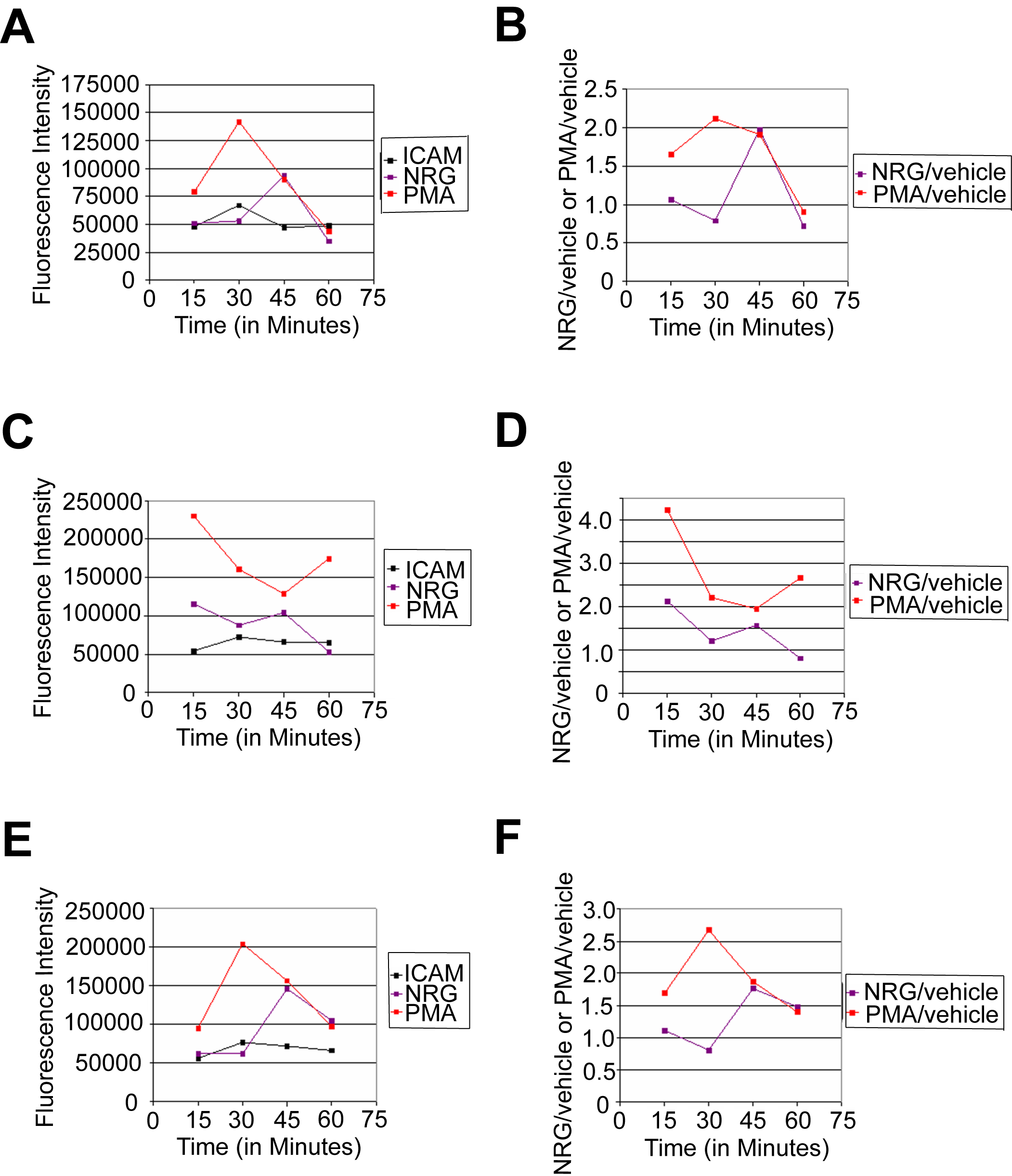

Supplement: Figure S1 — Variation of attachment induced by NRG1 and PMA. The NRG/ICAM and PMA/ICAM values were designed to isolate the effects of NRG1α or PMA on the cell adhesion state and thereby control for the vast array of other cellular and environmental processes that can impact adhesion. These data illustrate that the use of such ratios is a justified transformation of the data and that the variable adhesion states are not an artifact simply of changing ICAM-alone baseline adhesion. Displayed are raw fluorescence values of ICAM-alone, NRG, and PMA data from three experiments on different days using different cell lines. (A, C, E). These data show that although baseline adhesion shows little variation over time, NRG and PMA adhesion do considerably. The NRG/vehicle and PMA/vehicle ratios for those same three experiments also are shown next to their respective raw data (B, D, F). (0.85 MB TIF) [file pone.0001369.s001.tif]

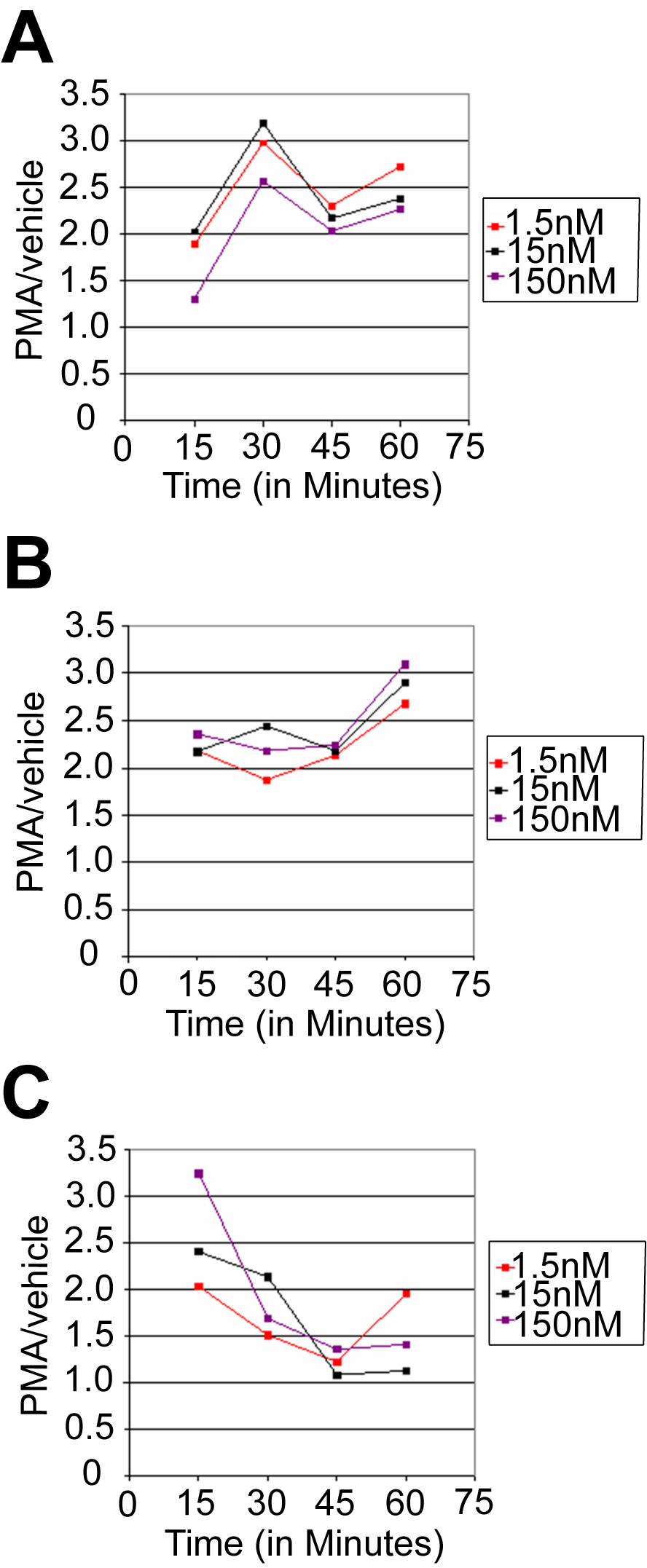

Supplement: Figure S2 — Varying adhesion patterns show individual consistency. Cells from three cell lines were subjected in the same experiment at the same time to three doses of PMA over two orders of magnitude (A-C). Wells from the three different cell lines were washed together at the same time and with the same technique using a 12-tip multi-pipettor. Within a cell line, the same general pattern of varying cell adhesion over time was conserved. However, the patterns of varying cell adhesion were quite different between cell lines with peaks and troughs occurring at different time points despite undergoing washings together at the exact same time with the multi-pipettor. This suggests that the response of cells from a particular cell line under the same conditions is relatively synchronized and that the varying adhesion observed is less likely to be simply noise. (0.34 MB TIF) [file pone.0001369.s002.tif]

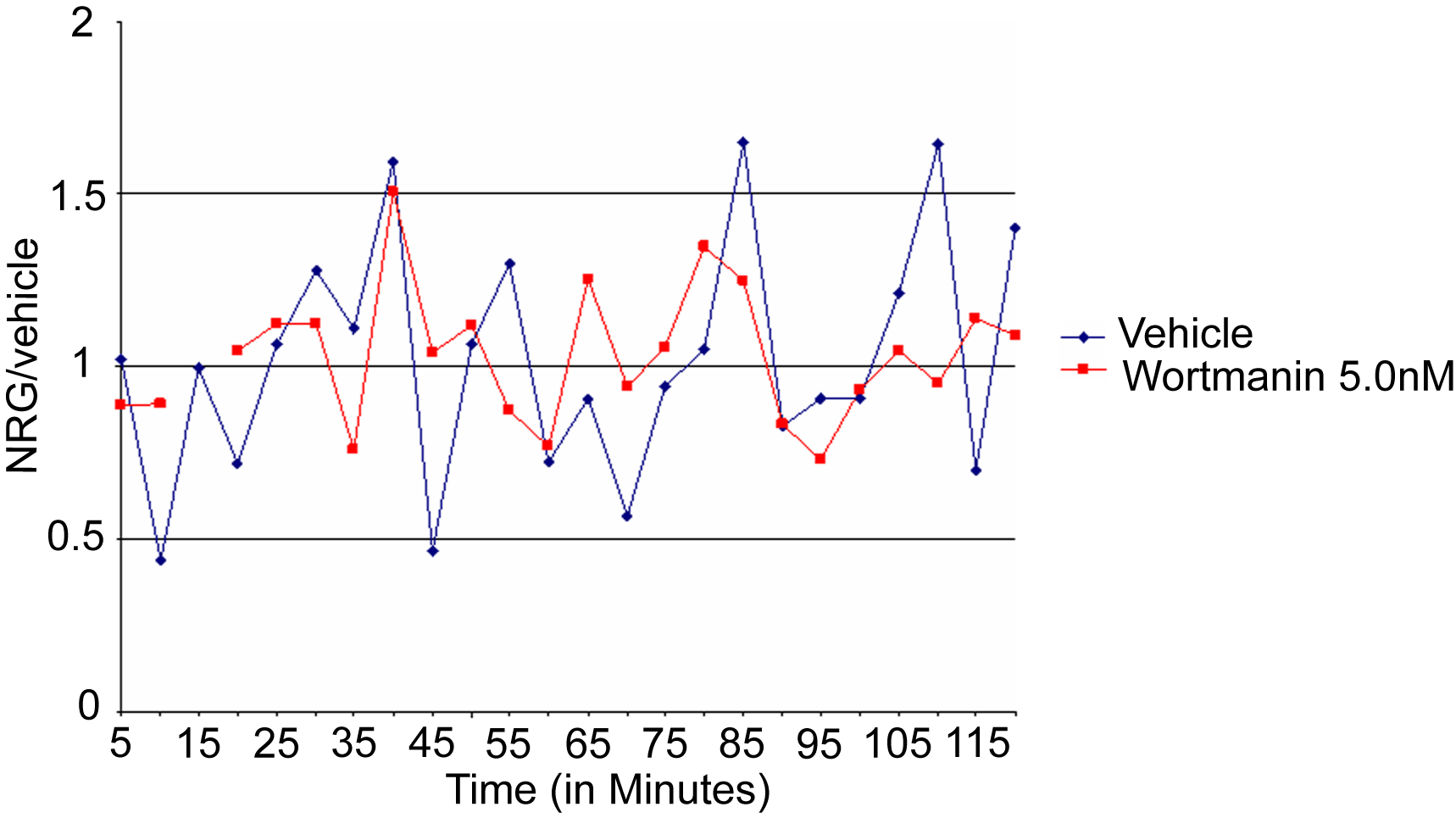

Supplement: Figure S3 — The MRVA appears intrinsic to a particular cell line and is dampened upon inhibition of involved signaling pathways. A representative example of one control cell line assayed at 5 minute time points with and and without PI3K inhibition by wortmannin. While there is no predictable pattern, the vehicle cells continue to recapitulate the same approximate peak and trough multiple times over the time course. The MRVA for the wortmannin-treated cells is substantially lower than the MRVA for cells treated only with DMSO vehicle (wortmannin MRVA: 0.779, vehicle MRVA: 1.211). As the cells can fluctuate over a 5 minute period from states of relatively strong net attachment to states of relatively strong net detachment, there is no obvious advantage to using 5 minute time points instead of 15 minute time points in order to help characterize the range of variability. All wells for each time point were washed at the same time and with the same technique using a 12-tip multi-pipettor. (0.33 MB TIF) [file pone.0001369.s003.tif]

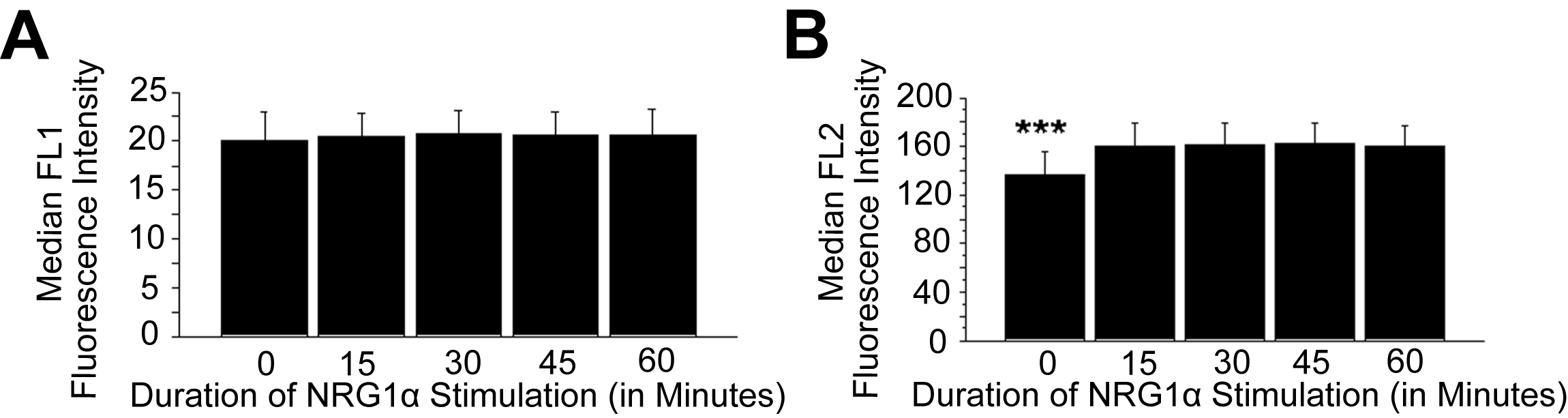

Supplement: Figure S4 — CD18, but not CD11a, integrin surface expression increases with NRG1α stimulation. Cells from 5 cell lines derived from patients with schizophrenia and 5 derived from normal controls were stimulated with NRG1α for 0, 15, 30, 45, or 60 minutes and then stained with anti-CD11a (αL) FITC-conjugated (A) or anti-CD18 (β2) PE-conjugated (B) antibodies and analyzed for expression by FACScan. The median fluorescence of 10 000 gated live cells was calculated for each cell line. Group data for both figures reflect the means of individual cell lines' median fluorescence values. *** represents p<0.001 and is the post-hoc value for every 2-way comparison with t = 0. (0.10 MB TIF) [file pone.0001369.s004.tif]
